# *ANXA11* mutations are associated with amyotrophic lateral sclerosis–frontotemporal dementia

**DOI:** 10.3389/fneur.2022.886887

**Published:** 2022-09-26

**Authors:** Yu Wang, Xiaohui Duan, Xiao Zhou, Renbin Wang, Xiangfei Zhang, Zhenhua Cao, Xiaoxia Wang, Zhi Zhou, Yu Sun, Dantao Peng

**Affiliations:** ^1^Department of Neurology, China-Japan Friendship Hospital, Beijing, China; ^2^Running Gene Inc., Beijing, China

**Keywords:** annexin A11, *ANXA11*, amyotrophic lateral sclerosis, frontotemporal dementia, genotype, phenotype [mesh]

## Abstract

**Background:**

The Annexin A11 (*ANXA11*) gene has been newly identified as a causative gene of amyotrophic lateral sclerosis (ALS) with or without frontotemporal dementia (FTD). The current study aimed to investigate the *ANXA11* mutations in a Chinese ALS–FTD or FTD cohort.

**Methods:**

We included ten probands/patients with suspected ALS–FTD or FTD. Mutational analysis of *ANXA11* was performed through Next Generation Sequencing (NGS) and Sanger sequencing. We collected and reviewed clinical presentation, neuropsychology test results, brain-imaging findings, and electrophysiological examination findings.

**Results:**

In total, six probands presented with ALS–FTD, and four with behavior variant FTD (bv-FTD). We identified a non-synonymous heterozygous mutation (c.119A>G, p.D40G) of *ANXA11* in proband 1, which is associated with ALS. However, this is the first report of the mutation causing ALS–FTD. Proband 1 started with abnormal behavior and progressed to classic upper motor nervous disease. Magnetic resonance imaging (MRI) showed significant bilateral temporal lobe atrophy and bilateral hyperintensities along the corticospinal tracts.18F-AV45-PET imaging showed negative amyloid deposits.

**Conclusion:**

*ANXA11*-related diseases have high clinical and genetic heterogeneity. Our study confirmed the contribution of *ANXA11* mutations to ALS–FTD. The *ANXA11* mutations established a complex genotype–phenotype correlation in ALS–FTD. Our research further elucidated the genetic mechanism of ALS–FTD and contributed to setting the foundation of future targeted therapy.

## Introduction

Amyotrophic lateral sclerosis, a lethal progressive neurologic disease, is characterized by selective degeneration of the lower and upper motor neurons. Approximately 5–10% of patients with ALS have a positive family history, suggesting that genetic factors substantially contribute to its pathogenesis. Frontotemporal dementia (FTD) is a spectrum of syndromes characterized by a progressive deterioration in behavior, personality, language, and cognition, associated pathologically with frontotemporal lobar degeneration (FTLD). ALS is closely related to FTD. Up to ~50% of patients with ALS show behavioral dysfunction and/or subtle cognitive impairment, while about 15% meet the psychiatry diagnostic criteria of FTD (termed as ALS–FTD) (1–3). A similar scenario is observed in FTD. Approximately 30% of patients with FTD have motor impairments, and 12.5% meet the diagnostic criteria for ALS (4, 5).

In the past few years, owing to the rapid development of next-generation sequencing, ALS–FTD-associated genes have been progressively identified. For example, mutations of *C9orf72, TARDBP*, and *TBK1* have been identified as major genetic causes of ALS–FTD. The aggregation of TAR DNA-binding protein 43 (TDP-43) in the affected brain regions and motor neurons is a common pathological characteristic of each of these variants (6–10) in up to 97% of ALS and 50% of FTD cases. Beyond that, mutations in *CCNF, CHCHD10, FUS, SQSTM1, UBQLN2*, and *VCP* are also associated with ALS–FTD (11). However, the genetic etiology of ALS–FTD in some patients remains unclear. In the current study, mutation in the Annexin A11(*AXAN11*) gene was proved to be linked to ALS–FTD in a Chinese clinical cohort. We also included a review of previously reported mutations with ALS or ALS–FTD in the *AXAN11* gene.

## Patients and methods

### Patients

In total, ten probands/patients with suspected ALS–FTD or FTD from the Department of Neurology, China–Japan Friendship Hospital in Beijing, were enrolled in the study from July 2019 to January 2022. The clinical characteristics, brain imaging results, and laboratory profiles were collected. This research was approved by the institutional board of the Ethics Committees of China–Japan Friendship Hospital in Beijing and followed the Declaration of Helsinki.

### Mutation analysis

Genomic DNA was extracted from peripheral blood samples collected from ten suspected patients and healthy volunteers, according to standard procedures. The repeat length of the pathogenic *C9orf72* GGGGCC repeat expansion was examined and excluded in these patients using polymerase chain reaction (PCR) amplification combined with microfluidic capillary electrophoresis.

Whole-exome sequencing was performed following the Illumina specifications. The isolated DNAs were firstly fragmented into 200–250 bp lengths by sonication. Then, DNA libraries were built using the KAPA Library Preparation Kit (Kapa Biosystems, KR0453) and sequenced *via* the Illumina Noveseq s4 platform (Illumina, San Diego, USA) with 150-bp paired-end reads. The human reference genome (UCSC hg19) was applied to the filter and aligned with the raw data using the Burrows-Wheeler Alignment tool (BWA-0.7.12, http://bio-bwa.sourceforge.net/). GATK software (www.broadinstitute.org/gatk) was used to identify single-nucleotide polymorphisms (SNPs), insertions, and deletions (indels). VEP [Ensemble Variant Effect Predictor, McLaren et al. (12)] was used to annotate all the variants, including the genetic position, type, allele frequency, conservation prediction, etc.

### Pathogenicity assessment

All the variants were filtered first against the 1,000 genomes project database, for a minor allele frequency (MAF) ≥ 1%, and ExAC hom AC ≥3. The obtained variants were further selected according to co-segregation, the genetic model, and an MAF <1% in three databases (1,000 genomes project_EAS, ExAC, and gnomAD_EAS). We then focused on analyzing variants of the ALS-related genes, which were included in the OMIM database. All the candidate pathogenic variants were confirmed by Sanger sequencing and classified according to the American College of Medical Genetics and Genomics (ACMG) standards (13). Finally, the *ANXA11* mutations were selected based on their clinical relevance and pathogenicity.

### Electrophysiological studies

For electrophysiological profiles, examinations were conducted using conventional equipment and according to the standard methods, with skin temperatures maintained between 32 and 34°C. Nerve conduction and needle electromyography (EMG) examinations were conducted on 10 patients.

### MR technique and protocol

All the patients underwent 3.0T MRI with a device using eight-channel head coils (Discovery MR750 scanner; GE Medical Systems, United States) in the China–Japan Friendship Hospital. The sequences performed included T1- and T2-weighted fluid-attenuated inversion recovery (FLAIR) and standard coronal T2-weighted sequences.

### 18F-AV45-PET examination

In total, five patients were selected for 18F-AV45 PET scans using the Discovery Elite scanner (GE Healthcare) at the Tiantan Hospital. 18F-AV45 PET was performed at 20 min and 50 min postinjection of 248 ± 58 MBq. 18F-AV45 PET profiles were analyzed using an ordered subset expectation maximization algorithm with weighted attenuation. Images were smoothed using a 5 mm Gaussian kernel with scatter correction and evaluated prior to the analysis of patient motion and adequacy of statistical counts. Finally, the standardized uptake value ratios (SUVRs) were computed and normalized according to the cerebellar gray matter reference region and the mean activity, from 50 to 70 min.

### Literature review

We searched and reviewed published reports of *ANXA11* mutations using PubMed. Clinical, biochemical, neuroimaging, and genetic data from individual references were sourced and compared with the corresponding results of our research.

## Result

### Clinical features

The current cohort included 10 patients with behavioral variant FTD (bv-FTD). In total, six had probable bv-FTD with ALS according to the Rascovsky criteria. The clinical characteristics of the current Chinese clinical cohort are displayed in Table 1.

**Table 1:** Clinical features of ten probands/patients.

	**Proband1**	**Proband2**	**Proband3**	**Proband4**	**Proband5**	**Proband6**	**Patient7**	**Patient8**	**Patient9**	**Patient10**
Age(y) at onset	66 Y	72 Y	68 Y	51 Y	61 Y	34 Y	70 Y	72 Y	78 Y	80 Y
Disease duration (months)	18 M	36 M	24 M	12 M	8 M	12 M	24 M	12 M	15 M	18 M
Gender (M/F)	F	M	M	M	M	F	M	M	F	F
Education (years)	9	6	9	12	6	16	12	9	6	2
Family history	Limb weakness (1 brother)	Limb weakness (1 brother)	Limb weakness (1 brother)	Limb weakness (1 brother)	Limb weakness (his mother)	Limb weakness (1 sister + her mother)	No	No	No	No
Cognitive sign	Behavioral executive deficits anomia	Behavioral executive deficits anomia	Executive deficits	Executive deficits	Executive deficits anomia	Executive deficits	Behavioral executive deficits anomia	Behavioral executive deficits anomia	Behavioral executive deficits anomia	Behavioral executive deficits anomia
MMSE	25	22	23	26	22	27	23	22	21	19
MOCA	21	19	20	22	20	25	19	20	19	18
DST-Forwards	7	8	7	8	8	8	7	7	7	7
DST-Backwards	5	5	5	6	5	6	5	6	5	5
VFT	20	19	21	49	21	50	45	43	30	21
TMT B-A time (second)	219	244	200	50	231	100	120	110	150	200
RAVLT LOT	30	34	31	39	29	40	42	39	41	40
RAVLT A30 min	10	10	10	11	11	12	10	9	8	9
BNT	20	18	22	25	21	24	23	21	20	20
StroopCWT	30	31	31	40	29	39	30	31	39	34
APOE with e4 allele	Negative	Negative	Negative	Negative	Negative	Negative	Negative	Negative	Negative	Negative
Site of onset	Bulbar + Upper limb	Upper limb	Upper limb	Upper limb	Upper limb	Upper limb + Lower limb	No	No	No	No
ALS clinical features	Dysphagia Dysarthria Limbs weakness Fasciculations Pyramidal signs	Dysarthria Limbs weakness Fasciculations Pyramidal signs	Dysarthria Limbs weakness Fasciculations Pyramidal signs	Limbs weakness Fasciculations Pyramidal signs	Dysarthria Limbs weakness Fasciculations Pyramidal signs	Dysarthria Limbs weakness Fasciculations muscluar atrophy Pyramidal signs	No	No	No	No
Needle EMG	Neurogenic lesion in the cervical, thoracic, and lumbosacral spinal cord	Neurogenic lesion in the cervical, thoracic, and lumbosacral spinal cord	Neurogenic lesion in the bulbar, cervical, thoracic, and lumbosacral spinal cord	Neurogenic lesion in the cervical, thoracic, and lumbosacral spinal cord	Neurogenic lesion in the cervical and thoracic spinal cord	Neurogenic lesion in the bulbar, cervical, thoracic, and lumbosacral spinal cord	Normal	Normal	Normal	Normal
Brain MRI	Bilateral temporal lobe atrophy	Bilateral frotal and temporal lobe atrophy	Bilateral temporal lobe atrophy	Left temporal lobe atrophy	Bilateral temporal lobe atrophy	Bilateral temporal lobe atrophy	Bilateral frotal lobe and left temporal lobe atrophy	Bilateral frotal and temporal lobe atrophy	Bilateral frotal lobe and right temporal lobe atrophy	Bilateral frotal and temporal lobe atrophy
18F-AV45-PET	Negative	Negative	N/A	N/A	N/A	N/A	Negative	N/A	Negative	Negative
Diagnosis	bv-FTD +ALS	bv-FTD +ALS	bv-FTD +ALS	bv-FTD +ALS	bv-FTD +ALS	bv-FTD +ALS	bv-FTD	bv-FTD	bv-FTD	bv-FTD
Gene	ANXA11 (c.119A>G,p.D40G)	N	N	N	N	N	N	N	N	N

MMSE, mini-mental state examination scale; MoCA, Montreal cognitive assessment scale; DST, Digit span test; VFT, verbal fluency test; TMT, trail making test; RAVLT, Rey auditory verbal learning test; BNT, Boston word naming test; Stroop CWT, Stroop color word test; ALS–FTD, behavioral variant frontotemporal dementia with amyotrophic lateral sclerosis; bv–FTD, behavioral variant frontotemporal dementia; N/A, not applicable; N, no pathogenic gene mutation was found; 18F-AV45-PET, 18F-florbetapir (AV45) positron emission tomography (PET) imaging.

**Figure 1:**
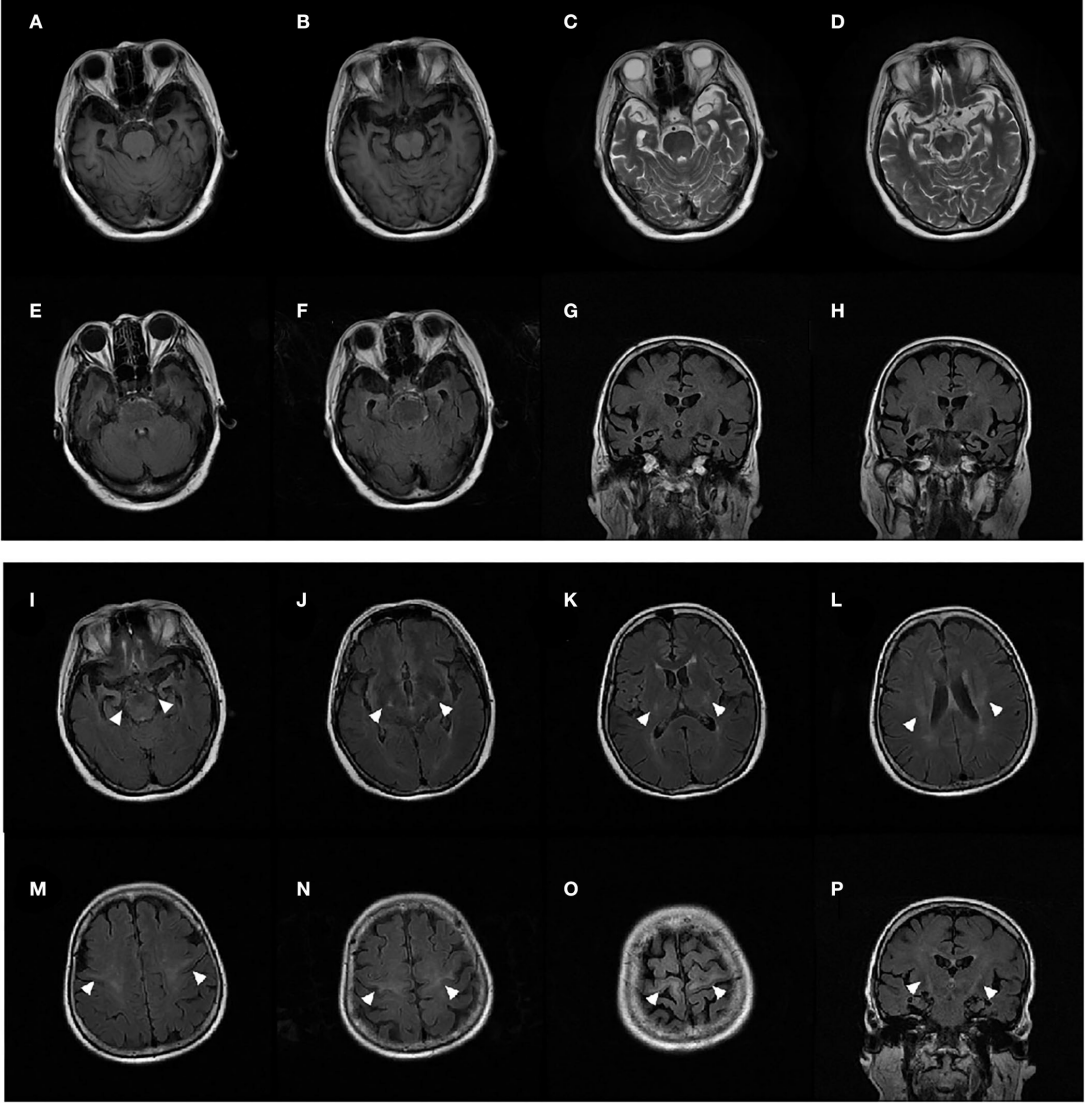
MRI of patient 1 showed significant bilateral temporal lobe atrophy **(A–H)**. T2-weighted fluid-attenuated inversion recovery (FLAIR) coronal and axial MRI displayed bilateral signal hyperintensities along the corticospinal tracts in the primary motor cortex, centrum semiovale, posterior limb of the internal capsule, and the cerebral peduncle **(I–P)** (arrows).

All 10 patients (6 men and 4 women) diagnosed with bv-FTD were from the Chinese mainland. The onset of symptoms occurred at the age of 34–80 years, median (IQR) is 69 (58.5–73.5). All 10 patients showed behavioral and executive deficits, and anomia. There were six patients with positive family histories. In total, one proband initially presented with dysarthria, and five probands presented with limb weakness as the initial symptom. Initially, Proband 1 presented with euphoria, loss of manners, impulsiveness, rash behavior, and difficulty cooking at the age of 66 years. A few months later, her speech became slurred, and she had difficulties in expressing and naming. The patient also had gradual weakness in both upper limbs. Fasciculations, hyperreflexia, and positive Babinski sign of the limbs were observed. EMG demonstrated a neurogenic lesion in the cervical, thoracic, and lumbosacral spinal cord. Brain MRI showed bilateral temporal lobe atrophy and bilateral signal hyperintensities along the corticospinal tracts (Figure 1). 18F-AV45-PET imaging showed negative amyloid deposits. The patient was diagnosed as having ALS with bv-FTD. She had an older brother who developed limb atrophy and weakness at 55 years of age and died at 67 years without providing a peripheral blood sample.

### *ANXA11* mutations and the updated genotype–phenotype spectrum

**Figure 2:**
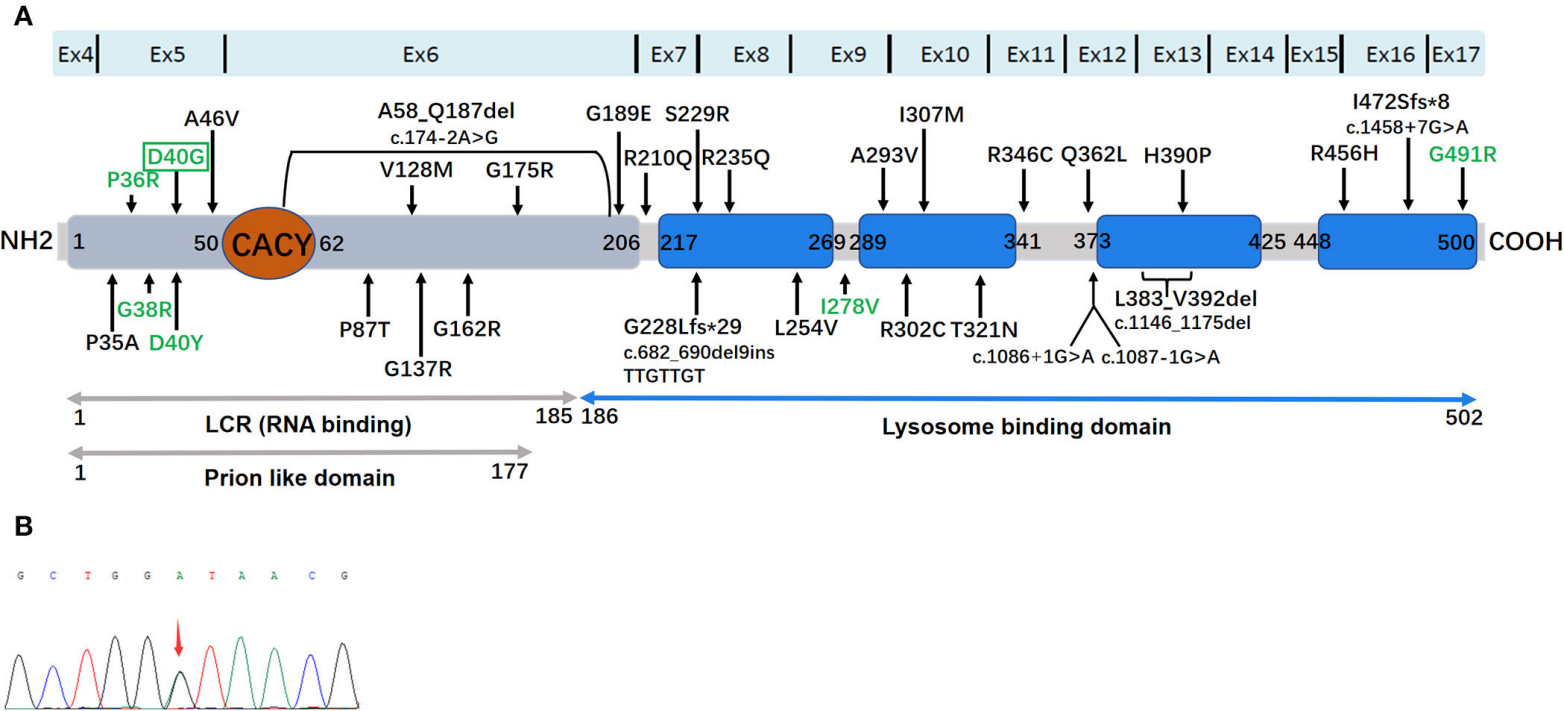
**(A)** The location and distribution of 32 mutations in a 2D schematic representation of the ANXA11 protein. ANXA11 protein and functional domains: a Prion-like domain (gray) was determined by the software PLAAC (Prion Like Amino Acid Composition). RNA (gray) and lysosome (blue) binding domains are represented. The binding site with Calcyclin/S100A6 (CACY) is located at N terminal (orange). The four highly conserved annexin domains are represented by blue square. Reported ALS-related mutations are displayed in black, and ALS/ALS-FTD-related mutations pointed by green. D4OG was detected in the present study are displayed in green by green box. **(B)** Sequence chromatograms of polymerase chain reaction (PCR) products show the heterozygous c.119A>G (p.D40G) mutation in this study.

We identified one non-synonymous heterozygous mutation (c.119A>G, p.D40G) in *ANXA11*, which was previously reported to be associated with ALS, but to our knowledge, this is the first time that has been found in ALS–FTD. By reviewing previous literature in the Human Gene Mutation Database (HGMD), we found out thirty-two different *ANXA11* variants have been identified in ALS and/or ALS–FTD, including patients from the United Kingdom, Southern Africans, Brazil, France, German, Korea, Spain, Japan, and China (Table 2) (14–26). To further investigate the correlation between phenotype and genotype, we reviewed and summarized all the studies on *ANXA11* mutations (Figure 2).

**Table 2:** Clinical and genetic characteristics of *ANXA11*-related diseases.

**Gene**	**Ethnicity**	**Nucleotide changes**	**Amino acid changes**	**Variants type/Zygo**	**Clinic features**	**References**
ANAX11	British	103C > G	Pro35Ala (P35A)	Missense (Het)	ALS	(14)
	Chinese, Korean	107C > G	Pro36Arg (P36R)	Missense (Het)	ALS, ALS-FTD	(15, 16)
	Euramerican, Korean, South African	112G > A	Gly38Arg (G38R)	Missense (Het)	ALS, ALS-FTD	(16–20)
	French, Brazilian	118G > T	Asp40Tyr (D40Y)	Missense (Het)	ALS, ALS-FTD, hIBM	(19, 21, 22)
	European, Chinese, Korean	119A > G	Asp40Gly (D40G)	Missense (Het)	ALS, ALS-FTD	(15–17), This study
	German	137C > T	Ala46Val (A46V)	Missense (Het)	ALS	(18)
	Chinese	174-2A > G	A58_Q187del	Canonical-Splice (Het)	ALS	(15)
	German	259C > A	Pro87Thr (P87T)	Missense (Het)	ALS	(18)
	Chinese	382G > A	Val128Met (V128M)	Missense (Het)	ALS	(15)
	Korean	409G > A	Gly137Arg (G137R)	Missense (Het)	ALS	(16)
	German	484G > A	Gly162Arg (G162R)	Missense (Het)	ALS	(18)
	British	523G > A	Gly175Arg (G175R)	Missense (Het)	ALS	(17)
	British	566G > A	Gly189Glu (G189E)	Missense (Het)	ALS	(17)
	French	629G > A	Arg210Gln (R210Q)	Missense (Het)	ALS	(19)
	Chinese	687T > A	Ser229Arg (S229R)	Missense (Het)	ALS	(15)
	Korean	c.682_690del9ins TTGTTGT	G228Lfs*29	Frameshift (Het)	ALS	(16)
	British	704G > A	Arg235Gln (R235Q)	Missense (Het)	ALS	(17)
	French	760C > G	Leu254Val (L254V)	Missense (Het)	ALS	(19)
	Spanish	832A > G	Ile278Val (I278V)	Missense (Het)	ALS-FTD	(23)
	Chinese	878C > T	Ala293Val (A293V)	Missense (Het)	ALS	(24)
	Chinese	904C > T	Arg302Cys (R302C)	Missense (Het)	ALS	(15)
	Chinese	921C > G	Ile307Met (I307M)	Missense (Het)	ALS	(24)
	Korean	962C > A	Thr321Asn (T321N)	Missense (Het)	ALS	(16)
	British	1036C > T	Arg346Cys (R346C)	Missense (Het)	ALS	(17)
	Taiwanese	1085A > T	Gln362Leu (Q362L)	Missense (Het)	ALS	(25)
	Japanese	1086 + 1G > A		Canonical-Splice (Het)	ALS	(26)
	German	1087–1G > A		Canonical-Splice (Het)	ALS	(18)
	Korean	1169A > C	His390Pro	Missense (Het)	ALS	(16)
	Chinese	1146_1175del	L383_V392del	Gross deletion (Het)	ALS	(15)
	Korean	1367G > A	Arg456His (R456H)	Missense (Het)	ALS	(16)
	Korean	1458 + 7G > A	I472Sfs*8	Splice (Het)	ALS	(16)
	Chinese	1471G > A	Gly491Arg (G491R)	Missense (Het)	ALS-FTD	(15)

ALS, amyotrophic lateral sclerosis; ALS–FTD, amyotrophic lateral sclerosis-frontotemporal dementia; hIBM, inclusion body myopathy; Het, heterozygous mutation.

## Discussion

Located on the human chromosome 10q22.3, the *ANXA11* gene encodes the 505 amino acid annexin A11 protein, which is a member of a calcium-dependent phospholipid-binding annexin protein family. The primary function of the annexin protein family is to bind Ca2+, RNA, other proteins, and lipid membranes. Unlike other family members, *ANXA11* shows a uniquely long N-terminal domain that contains the calcyclin binding site (residues 50–62). Calcyclin can mediate ubiquitination and proteasome degradation of many target proteins (27). In total, four conserved annexin domains, including annexin1-4, constitute the conserved C terminus (28).

*ANXA11*-related ALS was initially identified in 2017 by whole-exome sequencing in 180 sporadic-ALS (SALS) cases and 751 European familial-ALS (FALS) (17). Smith et al. identified six *ANXA11* mutations (G38R, D40G, G175R, G189E, R235Q, and R346C) in 9 patients from 6 families, and 3 SALS cases without FTD. In the aforementioned study, the D40G mutation was found to be the most common mutation. Patients carrying the D40G mutation presented a delayed-onset of classical ALS symptoms, with 5/6 cases having the bulbar-onset disease. Subsequently, a study in a non-Caucasian population supported the pathogenicity of D40G in the *ANXA11* mutation associated with ALS. Of note, a sporadic ALS case was found once in a Chinese mainland cohort of 383 patients with ALS or ALS–FTD (15). There was also another reported study of 500 Korean patients with SALS (16). Liu et al. failed to discover D40G; instead, they found two rare heterozygous missense variants, namely, c.878C>T (p.A293V) and c.921C>G (p.I307M), in another Chinese cohort with 434 patients with SALS and 50 patients who had the index FALS (24). If the results of the two Chinese cohorts are combined, the D40G mutation rate is rarely low (0.12%, 1/867) in the Chinese patients with ALS or ALS–FTD. The aforementioned results suggest that p.D40G mutation is not the primary cause of ALS in the Chinese population (24).

According to the functional analysis, p.D40G being located near the calcyclin-binding region could cause abnormal binding of calcyclin. Analyses from a postmortem p.D40G ALS case showed profuse annexin A11-positive aggregates in neurons and neuropil of the neocortex and hippocampus, and motor neurons of the spinal cord (17).

In the current study, patients with the same D40G mutation have different clinical symptoms: (1) five of six European patients and one Korean patient who carried the mutation initially showed difficulty in swallowing and speaking (bulbar-onset ALS) (17); (2) a Chinese patient initially displayed left arm weakness at the age of 59 years (15); (3) in the present study, proband 1 with the *ANXA11* p.D40G mutation initially presented abnormal behaviors, executive deficits, and anomia, and later progressed to classic upper motor nervous system damage in the bulbar and limbs. MRI showed significant bilateral temporal lobe atrophy and bilateral signal hyperintensities along the corticospinal tracts. The patient was diagnosed with ALS with bv-FTD. To our knowledge, this study is the first to associate the D40G mutation with ALS–FTD. Our results provided more genetic support for ALS and FTD.

Reviewing the literature, the spectrum of genotypes and phenotypes associated with *ANXA11*-related diseases has expanded as follows: (14–26) (i) late-onset or early-onset ALS (black mutations in Figure 2); (ii) ALS with FTD (P36R, G38R, D40Y, D40G, I278V, and G491R); (iii) inclusion body myopathy (hIBM), isolated or in combination with ALS/FTD (D40Y). In addition, the ordinary single nucleotide polymorphism (rs1049550, C>T, p.R230C, and MAF 0.44) in *ANXA11* may enhance the risk of sarcoidosis (29). Furthermore, the rs1049550T in the *ANXA11* allele plays a protective role for sarcoidosis in the Chinese Han nationality (30). Like other multisystem proteinopathies (MSP), *ANXA11*-related disorders possess a high clinical heterogeneity (Table 2), suggesting that diverse phenotypes driven by the *ANXA11* mutations require long-term patient follow-ups. Of the six mutations, four mutations that were related to the ALS–FTD phenotype were clustered in *ANXA11* within the long N terminus. The P36R, G38R, D40Y, and D40G mutations are near the calcyclin-binding domain in annexin 11, indicating the functional importance of this region. We know that calcyclin forms a regulatory complex with the calcyclin-binding protein (CACYBP) and RING-type E3 ubiquitin ligase SIAH-1, thereby regulating the ubiquitination and degradation of many proteins, including β-catenin (27). Therefore, calcyclin plays a critical role in proteostasis. However, the pathogenetic mechanism of *ANXA11* mutations leading to ALS–FTD is unclear. Teyssou et al. performed the neuropathological analysis for the G38R case and revealed that FTLD–TDP type A allocations were elicited by the deposition of a mass of TDP-43 lesions in the cortex (31). In patients with ALS, TDP-43 lesion allocations are common because it is associated with a pure FTD phenotype or behavior, related to non-fluent aphasia, or linked to the *GRN or C9orf72* mutation (32). Currently, *in vivo* and *in vitro* experiments are warranted to further this area of research.

In conclusion, this study confirmed the essential role of *ANXA11* mutations in ALS and ALS–FTD. Our results enhanced the understanding of the clinical spectrum and the underlying mechanisms of *ANXA11*-related diseases, including typical ALS, hIBM, FTD, and their combinations.

## Data availability statement

The datasets presented in this study can be found in online repositories. The name of the repository and accession number can be found at: National Center for Biotechnology Information (NCBI) BioProject, https://www.ncbi.nlm.nih.gov/bioproject/, PRJNA832024.

## Ethics statement

The studies involving human participants were reviewed and approved by the Ethics Committee of China-Japan Friendship Hospital (2021-1-Y0). The patients/participants provided their written informed consent to participate in this study.

## Author contributions

YW, XD, and DP designed the study. YW, XD, XZho, RW, and DP contributed patient material and clinical data. XW, ZC, XZho, ZZ, XZha, and YS carried out the experiments. YW, XD, DP, and RW analyzed and interpreted the data. YW and XD wrote the manuscript. All authors have made significant contributions and have approved the final version of this manuscript.

## Funding

This study received funding from Deutsche Herzstiftung e.V. The funder was not involved in the study design, collection, analysis, interpretation of data, the writing of this article or the decision to submit it for publication. All authors declare no other competing interests. Constanze Pfitzer is participant in the BIH Charité Clinician Scientist Program funded by the Charité—Universitätsmedizin Berlin and the Berlin Institute of Health. This work was supported by the Competence Network for Congenital Heart Defects (Federal Ministry of Education and Research/grant number 01GI0601) and the National Register for Congenital Heart Defects (Federal Ministry of Education and Research/grant number 01KX2140). We acknowledge financial support by Land Schleswig- Holstein within the funding program Open Access Publikationsfonds.

## Conflict of interest

Authors ZC and XW are employed by Running Gene Inc. The remaining authors declare that the research was conducted in the absence of any commercial or financial relationships that could be construed as a potential conflict of interest.

## Publisher's note

All claims expressed in this article are solely those of the authors and do not necessarily represent those of their affiliated organizations, or those of the publisher, the editors and the reviewers. Any product that may be evaluated in this article, or claim that may be made by its manufacturer, is not guaranteed or endorsed by the publisher.

## References

[1] Ringholz G Appel S Bradshaw M Cooke N Mosnik D Schulz P. Prevalence and patterns of cognitive impairment in sporadic ALS. Digest World Core Med J. (2006) 65:586–90. 10.1212/01.wnl.0000172911.39167.b6 16116120

[2] Wheaton M Salamone A Mosnik D Mcdonald R Appel S Schmolck H. Cognitive impairment in familial ALS. Neurology. (2007) 69:1411–7. 10.1212/01.wnl.0000277422.11236.2c 17909153

[3] Robberecht W Philips T. The changing scene of amyotrophic lateral sclerosis. Nat Rev Neurosci. (2013) 14:248–64. 10.1038/nrn3430 23463272

[4] Burrell J Kiernan M Vucic S Hodges J. Motor neuron dysfunction in frontotemporal dementia. Brain. (2011) 134:2582–94. 10.1093/brain/awr195 21840887

[5] Van Langenhove T Piguet O Burrell J Leyton C Foxe D Abela M. Predicting development of amyotrophic lateral sclerosis in frontotemporal dementia. J Alzheimers Dis. (2017) 58:163–70. 10.3233/JAD-161272 28387671

[6] Van der Zee J Gijselinck I Van Mossevelde S Perrone F Dillen L Heeman B. TBK1 mutation spectrum in an extended European patient cohort with frontotemporal dementia and amyotrophic lateral sclerosis. Hum Mutat. (2017) 38:297–309. 10.1002/humu.23161 28008748 PMC5324646

[7] DeJesus-Hernandez M Mackenzie I Boeve B Boxer A Baker M Rutherfoed N. Expanded GGGGCC hexanucleotide repeat in noncoding region of C9ORF72 causes chromosome 9p-linked FTD and ALS. Neuron. (2011) 72:245–56. 10.1016/j.neuron.2011.09.011 21944778 PMC3202986

[8] Renton A Majounie E Waite A Simón-Sánchez J Rollinson S Gibbs J. A hexanucleotide repeat expansion in C9ORF72 is the cause of chromosome 9p21-linked ALS-FTD. Neuron. (2011) 72:257–68. 10.1016/j.neuron.2011.09.010 21944779 PMC3200438

[9] Kabashi E Valdmanis P Dion P Spiegelman D McConkey B Velde C. TARDBP mutations in individuals with sporadic and familial amyotrophic lateral sclerosis. Nat Genet. (2008) 40:572–4. 10.1038/ng.132 18372902

[10] Sreedharan J Blair I Tripathi V Hu X Vance C Rogelj B. TDP-43 mutations in familial and sporadic amyotrophic lateral sclerosis. Science. (2008) 319:1668–72. 10.1126/science.1154584 18309045 PMC7116650

[11] Crook A Williams K Adams L Blair I Rowe D. Predictive genetic testing for amyotrophic lateral sclerosis and frontotemporal dementia: genetic counselling considerations. Amyotroph Lateral Scler Frontotemporal Degener. (2017) 18:475–85. 10.1080/21678421.2017.1332079 28585888

[12] McLaren W Gil L Hunt SE Riat HS Ritchie GR Thormann A. The ensembl variant effect predictor. Genome Biol. (2016) 17:122. 10.1186/s13059-016-0974-4 27268795 PMC4893825

[13] Richards S Aziz N Bale S Bick D Das S Gastier-Foster J. Standards and guidelines for the interpretation of sequence variants: a joint consensus recommendation of the American college of medical genetics and genomics and the association for molecular pathology. Genet Med. (2015) 17:405–24. 10.1038/gim.2015.30 25741868 PMC4544753

[14] Shepheard S Parker M Cooper-Knock J Verber N Tuddenham L Heath P. Value of systematic genetic screening of patients with amyotrophic lateral sclerosis. J Neurol Neurosurg Psychiatry. (2021) 92:510–8. 10.1136/jnnp-2020-325014 33589474 PMC8053339

[15] Zhang K Liu Q Liu K Shen D Tai H Shu S. ANXA11 mutations prevail in Chinese ALS patients with and without cognitive dementia. Neurol Genet. (2018) 4:e237. 10.1212/NXG.0000000000000237 29845112 PMC5963931

[16] Nahm M Lim S Kim Y Park J Noh M Lee S. ANXA11 mutations in ALS cause dysregulation of calcium homeostasis and stress granule dynamics. Sci Transl Med. (2020) 12:3993. 10.1126/scitranslmed.aax3993 33087501

[17] Smith B Topp S Fallini C Shibata H Chen H Troakes C. Mutations in the vesicular trafficking protein annexin A11 are associated with amyotrophic lateral sclerosis. Sci Transl Med. (2017) 9:eaad9157. 10.1126/scitranslmed.aad9157 28469040 PMC6599403

[18] Müller K Brenner D Weydt P Meyer T Grehl T Petri S. Comprehensive analysis of the mutation spectrum in 301 German ALS families. J Neurol Neurosurg Psychiatry. (2018) 89:817–27. 10.1136/jnnp-2017-317611 29650794

[19] Teyssou E Muratet F Amador M Ferrien M Lautrette G Machat S. Genetic screening of ANXA11 revealed novel mutations linked to amyotrophic lateral sclerosis. Neurobiol Aging. (2021) 99:102.e11–20. 10.1016/j.neurobiolaging.2020.10.015 33218681

[20] Nel M Mahungu A Monnakgotla N Botha G Mulder N Wu G. Revealing the mutational spectrum in southern Africans with amyotrophic lateral sclerosis. Neurol Genet. (2022) 8:e654. 10.1212/NXG.0000000000000654 35047667 PMC8756565

[21] Nunes Gonçalves J Leoni T Martins M Peluzzo T Dourado M Saute J. Genetic epidemiology of familial ALS in Brazil. Neurobiol Aging. (2021) 102:227.e1–4. 10.1016/j.neurobiolaging.2021.01.007 33618928

[22] Leoni T González-Salazar C Rezende T Hernández A Mattos A Neto A. A novel multisystem proteinopathy caused by a missense ANXA11 variant. Ann Neurol. (2021) 90:239–252. 10.1002/ana.26136 34048612

[23] Dols-Icardo O García-Redondo A Rojas-García R Borrego-Hernández D Illán-Gala I Muñoz-Blanco J. Analysis of known amyotrophic lateral sclerosis and frontotemporal dementia genes reveals a substantial genetic burden in patients manifesting both diseases not carrying the C9orf72 expansion mutation. J Neurol Neurosurg Psychiatry. (2018) 89:162–8. 10.1136/jnnp-2017-316820 28889094

[24] Liu X Wu C He J Zhang N Fan D. Two rare variants of the ANXA11 gene identified in Chinese patients with amyotrophic lateral sclerosis. Neurobiol Aging. (2019) 74:235.e9–12. 10.1016/j.neurobiolaging.2018.09.020 30337194

[25] Tsai P Liao Y Jih K Soong B Lin K Lee Y. Genetic analysis of ANXA11 variants in a Han Chinese cohort with amyotrophic lateral sclerosis in Taiwan. Neurobiol Aging. (2018) 72:188.e1–2. 10.1016/j.neurobiolaging.2018.07.002 30054183

[26] Sainouchi M Hatano Y Tada M Ishihara T Ando S Kato T. A novel splicing variant of ANXA11 in a patient with amyotrophic lateral sclerosis: histologic and biochemical features. Acta Neuropathol Commun. (2021) 9:106. 10.1186/s40478-021-01202-w 34099057 PMC8186038

[27] Fukushima T Zapata J Singha N Thomas M Kress C Krajewska M. Critical function for SIP, a ubiquitin E3 ligase component of the beta-catenin degradation pathway, for thymocyte development and G1 checkpoint. Immunity. (2006) 2:29–39. 10.1016/j.immuni.2005.12.002 16413921

[28] Wang J Guo C Liu S Qi H Yin Y Liang R. Annexin A11 in disease. Clin Chim Acta. (2014) 431:164–8. 10.1016/j.cca.2014.01.031 24508622

[29] Hofmann S Franke A Fischer A Jacobs G Nothnagel M Gaede K. Genome-wide association study identifies ANXA11 as a new susceptibility locus for sarcoidosis. Nat Genet. (2008) 40:1103–6. 10.1038/ng.198 19165924

[30] Feng X Zang S Yang Y Zhao S Li Y Gao X. Annexin A11 (ANXA11) gene polymorphisms are associated with sarcoidosis in a Han Chinese population: a case-control study. BMJ Open. (2014) 4:e004466. 10.1136/bmjopen-2013-004466 25056970 PMC4120255

[31] Mackenzie IR Neumann M Baborie A Sampathu DM Du Plessis D Jaros E. A harmonized classification system for FTLD-TDP pathology. Acta Neuropathol. (2011) 122:111–3. 10.1007/s00401-011-0845-8 21644037 PMC3285143

[32] Mackenzie I Frick P Neumann M. The neuropathology associated with repeat expansions in the C9ORF72 gene. Acta Neuropathol. (2014) 127:347–57. 10.1007/s00401-013-1232-4 24356984

